# Identification of a novel archaea virus, detected in hydrocarbon polluted Hungarian and Canadian samples

**DOI:** 10.1371/journal.pone.0231864

**Published:** 2020-04-17

**Authors:** János Molnár, Balázs Magyar, György Schneider, Krisztián Laczi, Sarshad K. Valappil, Árpád L. Kovács, Ildikó K. Nagy, Gábor Rákhely, Tamás Kovács

**Affiliations:** 1 Department of Biotechnology, Nanophagetherapy Center, Enviroinvest Corporation, Pécs, Hungary; 2 Biocentrum Ltd., Gyongyosoroszi, Hungary; 3 Institute of Medical Microbiology and Immunology, University of Pécs, Pécs, Hungary; 4 Department of Biotechnology, University of Szeged, Szeged, Hungary; 5 Institute of Biophysics, Biological Research Center, Szeged, Hungary; University of Trento, ITALY

## Abstract

Metagenomics is a helpful tool for the analysis of unculturable organisms and viruses. Viruses that target bacteria and archaea play important roles in the microbial diversity of various ecosystems. Here we show that *Methanosarcina* virus MV (MetMV), the second *Methanosarcina* sp. virus with a completely determined genome, is characteristic of hydrocarbon pollution in environmental (soil and water) samples. It was highly abundant in Hungarian hydrocarbon polluted samples and its genome was also present in the NCBI SRA database containing reads from hydrocarbon polluted samples collected in Canada, indicating the stability of its niche and the marker feature of this virus. MetMV, as the only currently identified marker virus for pollution in environmental samples, could contribute to the understanding of the complicated network of prokaryotes and their viruses driving the decomposition of environmental pollutants.

## Introduction

There is a recent trend in identifying viruses from metagenomic sequences [1, 2].

The metagenomic analysis is a useful tool to analyze uncultured prokaryotic abundance and monitor prokaryotic processes and interactions during bioremediation [3] and to optimize the biodegradation of pollutants [4].

It is important to know the dynamic changes of viral environments. Characterizing the virome of a metagenomic sample is frequently hindered by the phenomenon called viral dark matter, i.e. the fact that a high proportion (40–90%) of sequences from a metagenome cannot be aligned to any known organism [5]. Krishnamurthy and Wang identified three factors of viral dark matter: the divergence and length of virus sequences, the limitations of alignment based classification, and limited representation of viruses in reference sequence databases [5]. The value of searching publicly available microbial genome data for fragments of viral genomes has already been demonstrated [6]. These new viral genomes could serve as a useful resource for researchers as they explore the communities of viruses and microbes present in natural environments, the human body and in industrial processes.

Metagenomics analyses of bacterial and archaea viruses are carried out without culturing them. Therefore, their host, usually determined using a culture-based method, remains unknown. Several computational approaches exist to predict bacteriophage and archaea virus hosts, including the comparison of host and virus abundance profile, genetic homology, CRISPR analysis, exact matches of sequences between host and virus, and comparison of oligonucleotide profiles [7]. Each of these methods has disadvantages and their performance depends on several factors [7], such as metagenome composition, database content and host genome characteristics. Therefore, there is still no gold standard for computational discovery of hosts.

Phages and archaeal viruses play a crucial role in the dynamics of microbial diversity in various ecosystems [8–11], although little is known about these processes. Similar viruses are often highly abundant in similar ecosystems, for example, the crAssphage in human fecal samples [12]. Recently, it was proven that the crAssphage has co-evolved with the human gut microflora over millions of years [13]. In order to identify an abundant archaea virus in hydrocarbon polluted samples, we analyzed metagenomic data from hydrocarbon-contaminated soil. Analysis of an abundant virus genome could facilitate insight into the archaea host-virus relationship, including the ecological role of viruses in complex ecosystems, contributing to a better understanding of the processes that lead to the bioremediation of hydrocarbon pollution.

## Material and methods

### Site sampling

Sampling was based on the order (number 4184-20/2015) of the Government Office of Baranya County. The GPS coordinates of the sampling sites were: B1: 46°22'37.1"N 17°53'52.1"E, B2: 46°22'36.6"N 17°53'48.4"E, B3: 46°22'35.5"N 17°53'49.3"E and B4: 46°22'36.5"N 17°53'51.4"E. The sampled location was used as a military base and airport of the Hungarian army between 1936–1995 and by the US forces between 1995–2004. B1 was located directly at a damaged underground gas oil tank, B2 approximately 10 m from B1, B4 at a damaged gas oil pipeline, and B3 was drilled outside of the military base fence as a control. Samplings were carried out with a Borro driller, diameter 65 mm, and samples were taken from each hole at three depths: 0.8–1.0m, 5.0–5.5m and 7.0–7.5 m. Samples were stored in sealed glass bottles at +4 °C until analysis.

### DNA isolation and sequencing

DNA isolation occurred in three parallels from each sample using the PowerSoil Max kit (Mo Bio Laboratories Inc., USA) following the protocol provided by the producer. DNA library was prepared with Nextera XT DNA Library Prep kit (Illumina Inc., USA) and the library sequenced paired-end on an Illumina MiSeq next-generation sequencer (Illumina Inc., USA) using a V3 600 cycles kit (Illumina Inc., USA), according to the protocol of the kit producers.

### Analysis of TPH concentration

TPH concentration was analyzed with a CVH InfraCal TOG/PTPH Analyzer which, was calibrated in the 125–2000 ppm range.

### De novo assembly and annotation

Next-generation reads were analyzed for quality using the FastQC program (version 0.11.5) with default parameters. Low-quality bases and reads were trimmed and/or removed using the Trim galore (version 0.4.4 with paired mode) and Trimmomatic (version 0.36 with paired mode and using the CROP:150 MINLEN:150 parameters) programs [14]. The quality filtered reads were assembled by the MyPro software package [15]. In the assembly process, Assembly.py (using used Abyss, Soap, Spades and Velvet assemblers) and Integrate.py python scripts were used. Assembled contigs were annotated by Prokka version (1.12) [16] and blasted against the NCBI nt database [17].

During the downstream analysis, an assembled unknown 67,826 nucleotide long sequence, a potential archaeal virus genome, was used as a reference. The quality trimmed reads were aligned to this reference sequence with the bwa mem program (version 0.7.12-r1039) with default settings and paired mode [18]. Redundant reads were removed by samblaster [19] (version 0.1.24) and the filtered reads were sorted by coordinates with samtools (version 0.1.19-96b5f2294a) [20].

### Host prediction

CRISPR spacer sequences were downloaded from CRISPRdb [21] (Last updated 2017-05-09). The spacer sequences were mapped by the bwa aln program [22] with default settings to the de novo assembled hypothetical virus sequence.

The abundance profiles of bacteria in the analyzed samples were calculated by the Centrifuge program version 1.0.3-beta [23] using paired-end mode and default settings with a pre-built database which contained bacterial, archaeal, viral and human genome sequences. It was downloaded from the authors website ftp://ftp.ccb.jhu.edu/pub/infphilo/centrifuge/data/p_compressed+h+v.tar.gz (Last updated 12/06/2016).

Tetramer nucleotide abundance values were calculated using the Jellyfish program [24]. The *Methanosarcina barkeri str*. *Fusaro* reference sequence was downloaded from NCBI (Accession ID: NC_007355.1) and used for the calculation. The parameters were–m 4 searching for tetramers and using hash size–s 4560M. The same parameters were used for the MetMV virus sequence. The shuffleseq program from the EMBOSS software suite [25] was used to generate randomly shuffled sequences from the aforementioned *Methanosarcina* genome sequence.

### Occurrence of MetMV in the samples

Analyzing the samples, the quality-trimmed reads were mapped back to the de novo assembled MetMV virus contig and the mapped reads were counted by the samtools idxstats tool. The number of mapped reads were normalized for the fastqc library size. The average was calculated from the normalized results of the three DNA samples originating from the same soil sample.

Additionally, in the analysis of the SRA samples, the percentage of the covered bases of the sequence was counted. It was previously shown that this could be used to indicate the viral presence in metagenomic samples [26, 27]. A 75% genome coverage cut-off value was used for the viral presence [27].

## Results

### De novo assembly, annotation

Next generation reads were generated from the samples using the Illumina Miseq platform. During the sequencing process, we obtained from 64,268 to 2,739,140 raw and from 50,323 to 2,395,890 high quality trimmed paired-end reads indicating the loss of a small fraction during the quality filtering process. From this data, different complement analyses were carried out to find specific viruses from the hydrocarbon contaminated soil. In this habitat, microorganisms whose viruses have not been well characterized can be found, especially anaerobic archaeal lifeforms. These viruses could potentially be used as indicators for those archaea which often difficult to detect with classical biological methods. Initially, the next generation reads were assembled with *de novo* assembler methods from DNA samples originating from deep (7.5–8.5 m) soil samples and contaminated with hydrocarbon pollutant.

**Fig 1:**
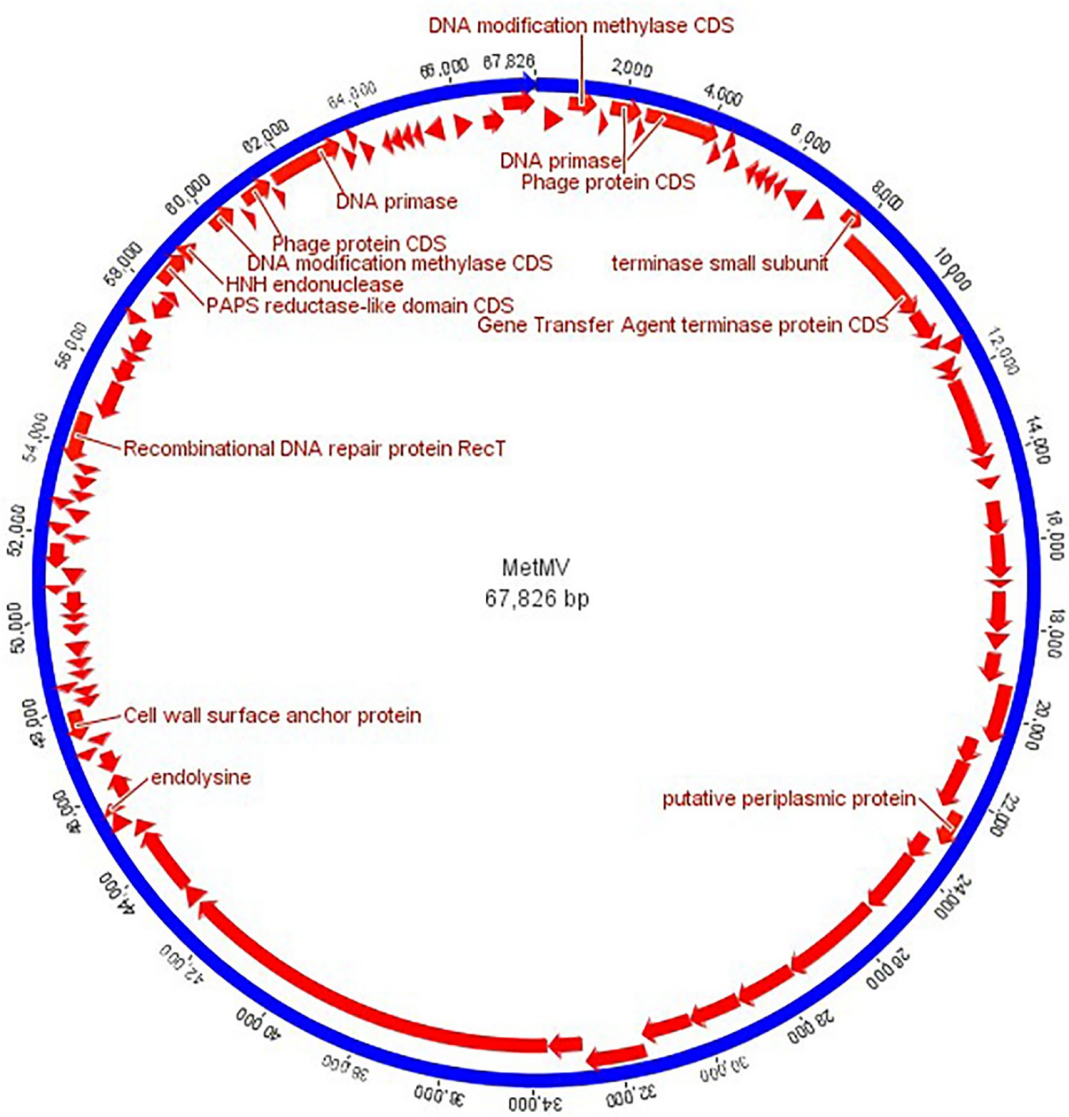
Map of the MetMV virus genome. The genome is circular, 67,826 bp long. Arrows represent the predicted open reading frames (ORFs) and arrowheads show the direction of transcription. Annotated functions other than hypothetical proteins are predicted.

Further analysis was carried out on contigs longer than 60 kbp. A 67,826 bp long contig was selected from a sample obtained at a depth of 7.5 m at the B1 location. It was searched against the NCBI viral and full nucleotide collection, but no similarity was found with the database sequences. The genome of the newly discovered virus (named MetMV) was circular and 8,697 bp direct terminal repeats were detected (Fig 1).

To measure the abundance of the hypothetical virus (MetMV) in the samples, the trimmed reads were mapped back to the generated *de novo* sequence. Abundances from the number of mapped reads were counted by normalizing for the sequencing library size. The presence of this virus was detected in four samples, where the percentage of viral mapped reads was between 0.1 and 8.4 (Table 1).

**Table 1:** Occurrence of MetMV virus. Sample names shows the location (B1-4) and depth of sampling. Threshold of MetMV presence was 0.01% and for TPH pollution 125 ppm.

Sample name	Sample accession number	Percentage of reads mapped to MetMV (%)	MetMV presence (1 = yes, 0 = no)	TPH pollution (1 = yes, 0 = no)
B1 1.0m	SAMEA4368876	0.0	0	0
B1 5.8m	SAMEA4368878	0.0	0	0
B1 7.5m	SAMEA4368881	8.4	1	1
B2 1.0m	SAMEA4373315	0.0	0	0
B2 5.5m	SAMEA4373317	0.0	0	0
B2 7.5m	SAMEA4373321	0.8	1	1
B3 1.0m	SAMEA4373324	0.0	0	0
B3 5.5m	SAMEA4373325	0.0	0	0
B3 7.8m	SAMEA4373328	0.0	0	0
B4 0.8m	SAMEA4373332	0.0	0	0
B4 5.5m	SAMEA4373334	0.0	0	0
B4 7.5m	SAMEA4373336	0.1	1	1

Additionally, in the Prokka annotation, only hypothetical proteins from the predicted 39 open reading frames (ORFs) were found. A function was assigned to 14 ORFs with manual curation (Fig 1).

### Host prediction

The relationship between archaeal viruses and their unknown hosts is often difficult to determine using laboratory methods as these microorganisms are typically hard to grow in an artificial *in vitro* system. On the other hand, when non-cultivation-based methods are used, various *in silico* tools are available for estimation of the host for isolated archaeal virus [7].

Initially, a specific CRISPR spacer sequence which could be found in the virus was identified from the CRISPR database [21]. These spacer sequences were aligned to the MetMV virus genome to search for similarities. In the alignment, five hits were identified from four different spp. Two strong candidates for hosts were chosen, *Methanosarcina mazei* S-6 and *Methanocaldococcus vulcanius* M7 which were anaerobe methanogen archaea. The MetMV virus abundance was calculated from the aligned reads and compared to *Methanosarcina* sp. homologue reads, which were identified using the Centrifuge classification engine [23]. Both the *Methanosarcina* sp. raw read abundance and library size normalized abundance (raw read abundance divided by the number of total reads in the analyzed samples, expressed as a percentage) were calculated and compared to the MetMV raw read and library size normalized abundance values. Strong correlations (0.979 for the raw read numbers and 0.980 for the library normalized values) were detected in case of *Methanosarcina* sp. Additionally, a third method was applied for host prediction analysis, the k-mer frequency of genome sequences. The Jellyfish program [24] and tetramer oligonucleotide frequencies were used. A series of frequency data were calculated for the MetMV and for the *Methanosarcina barkeri* str. Fusaro and three further frequency datasets were generated using shuffleseq [25] for negative controls from the same *Methanosarcina* sp. genome but nucleotide order of the sequence was randomly shuffled. This negative control provided the same length and 1-nucleotide frequency (content) as the investigated genome. However, tetramer frequencies were altered (in a random manner). In the case of real data and the average value of three randomly shuffled sequences (negative control), correlations between data for *Methanosarcina* sp. and MetMV virus were 0.66 and 0.5 respectively.

All three independent methods showed a strong correlation between the *Methanosarcina* genus and the MetMV virus genome. These results (Table 2) strengthened the hypothesis that MetMV’s host could be grouped into the *Methanosarcina* genus. It should be noted that MetMV’s G+C content (42.7 mol%) was slightly above that of *Methanosarcina barkeri* str. Fusaro’s (39.3 mol%). However, these data were close enough to indicate that a *Methanosarcina* sp. could be the host of MetMV [28].

**Table 2:** Summary of MetMV’s host prediction.

Predicted genus based on CRISPR	Abundance correlation analysis		k-mer frequency correlation analysis	
	Raw	Normalized	*Methanosarcina* sp. and MetMV	Control
*Aeropyrum* sp.	-0.05	-0.06	n.d.	n.d.
*Methanocaldococcus* sp.	0.05	0.04	n.d.	n.d.
*Methanosarcina* sp.	0.98	0.98	0.66	0.50
*Tannerella* sp.	-0.05	-0.16	n.d.	n.d.

n.d.: not determined. Raw and normalized abundance correlation analysis and control of k-mer frequency correlation analysis are defined in the Methods section.

### Co-occurrence with hydrocarbon pollution

The occurrence of the MetMV virus was investigated in metagenomes originating from all the samples. No significant correlation could be detected between the TPH concentration of the samples and the proportion of reads which could be mapped to the MetMV. However, if samples were marked with 1 when polluted with TPH (above 125 ppm, the lowest value of the calibration for the TOG/TPH analyzer) or with 0 when non-polluted, and marked with 1 when the MetMV virus could be detected (above 0.01% of the total reads in the sample) or with 0 when absent (Table 1), a significant correlation (1.0) was detected. These results suggest that there is no direct correlation between the concentration of TPH pollution and MetMV abundance, however, this virus tends to occur in TPH polluted samples. To test this hypothesis, the NCBI SRA database was searched for whole metagenome sequences originating from oil-contaminated samples sequenced on Illumina platforms. Bioproject PRJNA183510 was used, containing data generated from samples collected from hydrocarbon contaminated sites in Canada. MetMV was present in all samples from the MHGC oil field, and in 5 of the 6 Suncor tailing pond metagenomes (using the same threshold as applied to samples from the Taszar military base). Though no direct analysis was available, these samples were probably TPH contaminated as the MHGC oil field is an oil reservoir [29] and the Suncor tailing ponds contained high amounts of hydrocarbons and the mature fine tailings remained static for nearly 35 years [30]. It should be noted that MetMV could not be detected in many other metagenomes of this Canadian study, comprising Illumina WGS metagenome investigations of 93 samples [31].

A recent study characterized a *Methanosarcina*-infecting virus (MetSV), providing its 11 kb long complete genome sequence [32]. Here, we mapped reads originating in this study and from the Bioproject PRJNA183510 against the MetSV genome sequence. However, the virus could not be detected using the threshold of 0.01% of the total reads. This did not exclude the presence of this virus in the investigated samples because its short genome (compared to the MetMV’s genome size) means that the number of mapped reads may not reach the threshold even when the abundance of MetSV is similar to MetMV.

## Discussion

A 67,826 bp long circular complete genome of a virus (named MetMV) was assembled from reads occurring with high frequency in a metagenome sequenced from a hydrocarbon polluted soil sample. The small proportion of annotated ORFs could be due to the viral dark matter, i.e. the fact that reads originating from a metagenome sequencing could not be assigned to any known organism [5], indicating that there is a significant proportion of still unknown sequences and organisms. Since no cultivation of the novel virus was possible due to lack of its isolation, a cultivation-free *in silico* approach was chosen to predict its host. We used different bioinformatics prediction methods to achieve the best possible result to connect the host and its virus. Because the CRISPR system binds host and virus together, it was a good method to search for historical events connected to viruses in the host genome. This prediction may be more reliable than those derived from other methods as it showed co-occurrence and was based on the hypothesis of co-evolution of bacteria/archaea and virus. To strengthen the result of this method, the other co-occurrence methods and tetranucleotide frequency were used in parallel, as each of these methods investigated different features of the metagenome, archaea and virus genome.

A *Methanosarcina* sp. archaea was predicted as host of MetMV using *in silico* tools. It should be highlighted that *Methanosarcina* sp. plays an important role in the bioremediation of hydrocarbon polluted soil [33].

Prokaryotic viruses are essential in driving processes in microbial ecosystems and maintaining fluctuating selection [11]. Host-virus interactions can be antagonistic or mutualistic, i.e. prokaryotic viruses are not only predators, but they contribute to genetic variability, development of new ecotypes thus enabling colonization of new niches or even protection and maintenance of ecotypes [34–36]. Phages contribute to functional stability in microbial communities [37].

Methanogenesis is the process when anaerobic archaea convert small organic molecules (CO_2_, acetate, methylamine, formate etc.) to methane [38]. Members of the Methanosarcinales order are functionally important players in methanogen communities because they are highly diverse and some of them are capable of more than one methanogenesis pathway [39].

Because prokaryotic viruses can act also at the sub-species level, influencing the metabolic properties of the clades, thus driving the formation of new ecotypes, as it was proven in coevolution experiments [40], we can assume that viruses of Methanosarcinales can contribute to the selection of the methanogenesis pathway(s) used in the given ecosystem. Several, even competing, processes may occur under methanogenic conditions, like methanogenesis and sulfate reduction, which could co-exist on shared substrates [41]. It may be hypothesized that prokaryotic viruses contribute to the “fine tuning” these processes. To do this, viruses can sense host cell abundance, either directly, as observed with the PBdelta group infecting *Bacillus* sp., which produces a peptide under lytic conditions and releases it into the environment [42], or indirectly, via multiplication of infection (host abundance makes it more likely to encounter its phages). Recently, it was demonstrated that soil viruses have potential to influence host metabolism and community structure [43]. Based on the high abundance of MetMV in the investigated samples, we hypothesize that this virus may play an important role in the local ecosystem. However, whether MetMV contributes to the selection of methanogenesis pathways, to “fine tuning” of competing metabolic processes or to the adaptation, niche colonization or even niche protection of its host, remains to be determined in later research.

## Conclusions

Complete genome of the virus MetMV was assembled from reads originating from metagenome of a hydrocarbon polluted soil sample. A *Methanosarcina* sp. was predicted as host of MetMV and MetMV is the second *Methanosarcina* sp. virus whose complete genome has been determined until now. Occurrence of MetMV strongly correlates with the presence of hydrocarbon pollution in environmental samples.

Recently, it was found that crAssphage’s genome structure remained conserved in the stable environment of the human gut, possibly indicating the stability of this niche [13]. crAssphage can be used as a marker virus of human fecal contamination [13]. Similarly, MetMV’s high abundance in samples taken from distant geographic locations could indicate the high stability of this niche and the marker feature of this virus. These findings support the view that prokaryotes with their viruses should be regarded as holobiont contributing to diversification and accommodation to environmental changes via ecological speciation [11].
